# Effects of grassland degradation on soil ecological stoichiometry and soil microbial community on the South of the Greater Khingan Mountains

**DOI:** 10.3389/fmicb.2024.1438787

**Published:** 2024-11-18

**Authors:** Yuyu Li, Lixing Zhao, Mian Gul Hilal, Lizhu Guo, Yandong Zhang, Yu Ji, Xiaowei Jiang, Lifen Hao, Kejian Lin

**Affiliations:** ^1^Institute of Grassland Research, Chinese Academy of Agricultural Science, Hohhot, China; ^2^Key Laboratory of Biohazard Monitoring, Green Prevention and Control for Artificial Grassland, Ministry of Agriculture and Rural Affairs, Hohhot, China; ^3^Institute of Agricultural and Animal Husbandry of Hinggan League, Ulanhot, China

**Keywords:** grassland degradation, soil properties, soil enzyme activity, soil metals elements, soil microbial communities

## Abstract

Grassland which covers 40% of terrestrial land is an important ecosystem having a multitude of functions, which has suffered various degrees of degradation with the interaction between global climate change and unreasonable human utilization (e.g., grazing and reclamation). Improved understanding of soil and microbial community diversity during meadow steppe degradation is crucial for predicting degradation mechanisms and restoration strategies. Here, we used Illumina sequencing technology to investigate the patterns of soil microbial community structure and the driving factors of its change across different degradation degrees of meadow steppe [i.e., non-degraded grasslands (NDG), lightly degraded grasslands (LDG), moderately degraded grasslands (MDG), and severely degraded grasslands (SDG)] south of the Greater Khingan Mountains. Our results showed a significant variation in soil properties, enzyme activity, and soil metal elements across the degraded meadows. Soil available phosphorus (AP), urease (UE), and cellulase (CL) in soils increased with the intensity of grassland degradation. Grassland degradation significantly decreased soil bacterial and fungal richness. In addition, grassland degradation significantly increased the relative abundance of Firmicutes (from 1.65% to 5.38%) and Myxococcota (from 2.13% to 3.13%). Degradation considerably increased the relative abundance of Ascomycota (from 66.54% to 75.05%), but decreased Basidiomycota (from 18.33% to 9.92%). The relative abundance of nitrogen fixation and cellulolysis decreased significantly due to grassland degradation. For fungal functional guilds, the relative abundance of pathotrophs increased while saprotrophs decreased significantly with increasing severity of degradation. Total nitrogen (TP), AP, available potassium (AK), manganese (Mn), lead (Pb), UE, sucrase (SC), and alcalase protease (ALPT) were the main drivers of soil bacterial community composition, while TP, AP, AK, Pb, UE, and SC were the main drivers of soil fungal community composition in the degraded grassland. Our findings demonstrated that severe grassland degradation has an enormous effect on soil microbial communities and soil physicochemical dynamics. These findings improve our theoretical understanding of the interactions between soil microbial populations and soil environmental variables in degraded grassland.

## Introduction

Grasslands are one of the largest ecosystems in the world, which is of great significance to the stability and balance of the Earth's biosphere and the sustainable development of human society (Bardgett and van der Putten, 2014). China has 400 million hectares of grassland, accounting for more than 40% of the land area (Li et al., 2023). The meadow steppe transition zone in the South of the Greater Khingan Mountains is a large ecotone in northern China with significant ecological and environmental importance (Jin et al., 2024). In recent decades, increased human activities (e.g., grazing and reclamation) and climate warming (Miguel et al., 2020; Dong et al., 2020) have led to the loss of productivity and degradation of the ecological functions of the meadows in the transition zone (Zhou et al., 2019; Dong et al., 2020). The degradation of ecological functions poses a serious threat to the sustainable development of the environment and the economy of the region. Recently, the local government identified it as the core ecological problem (Xue et al., 2019).

Grassland degradation is a long and complicated process (Li et al., 2023), not only influences in plant composition, diversity, and aboveground and underground biomass (Peng et al., 2020a), but also affects soil structure and nutrient loss (Wang et al., 2020). Previous studies have predominantly concentrated on studying the dynamics of plant communities, including cover, biomass, and diversity, as well as soil characteristics, such as soil organic carbon and nutrients (Li et al., 2021b; Kotzé et al., 2017). However, such studies rarely considered grassland soil microorganisms, important components of the grassland ecosystem, and drive biogeochemical cycles and energy flow (Murugan et al., 2014; Legay et al., 2014).

Degradation of grasslands can disrupt the balance between the nutrients required by soil microbes and the availability of nutrients in the environment, changing the structure and diversity of microbes (Li et al., 2023; Luo et al., 2023; Gao et al., 2023). A previous study suggested soil pH is an important environmental factor regulating soil microbiome (Luo et al., 2020). Particularly, increased grassland degradation leads to an elevated soil pH (Su et al., 2006), resulting in a decline in the diversity of bacteria and fungal (Nian et al., 2024). Besides, disturbance and soil nutrients (e.g., carbon and nitrogen) can have a significant effect on microbial communities and their abundance (Ye et al., 2024). For instance, grassland degradation negatively impacts soil carbon and nitrogen content in the grasslands on the Loess Plateau (Luo et al., 2020), decreasing soil microbial biomass, and abundance (Zhou et al., 2019). Jiang et al. (2024) found that as the degradation intensified, grassland vegetation characteristics reduced significantly, and the diversity and composition of microflora varied significantly among all degraded meadows.

Previous studies have also demonstrated that soil trace metal elements play a pivotal role in elucidating the structure of soil microbiomes and the functioning of ecosystems (Dai et al., 2023). For example, Fe and Mn are essential for microbial respiration, Cu and Zn are crucial for immunocompetence, and Fe and Ni are vital for N fixation (Feng et al., 2019). Therefore, establishing the relationships between soil environmental factors and the structure and diversity of microbial communities is essential to elucidate the underlying mechanisms by which grassland degradation affects soil microbial dynamics. Furthermore, understanding the response of the microbial community to grassland degradation can help us better understand, predict, and manage grassland ecosystems in degraded.

Thus, the primary objective of this study was to (1) Evaluate the responses of soil environmental factors to the degradation of grasslands at different gradients; (2) Clarify the microbial response to the degradation of grasslands at different gradients; and (3) investigate the interactions between soil environmental factors and soil microbiome to the degradation of grasslands at different gradients.

## Materials and methods

### Study region

This study was conducted in August 2022 in the meadow grassland of southern Greater Khingan (average altitude of 900 m asl), located at the intersection of Greater Khingan, Horqin Grassland, and Xilinguole Grassland. It is ~400 km long in the east and west and 280-km wide in the south and north. The region experiences a frost-free period of 80–90 days, an annual average air temperature of 1°C, an annual average accumulated temperature of 1,800–2,000°C, and an annual average precipitation of 370–380 mm. These grasslands are grazed from June to November each year.

### Sample collection

**Figure 1:**
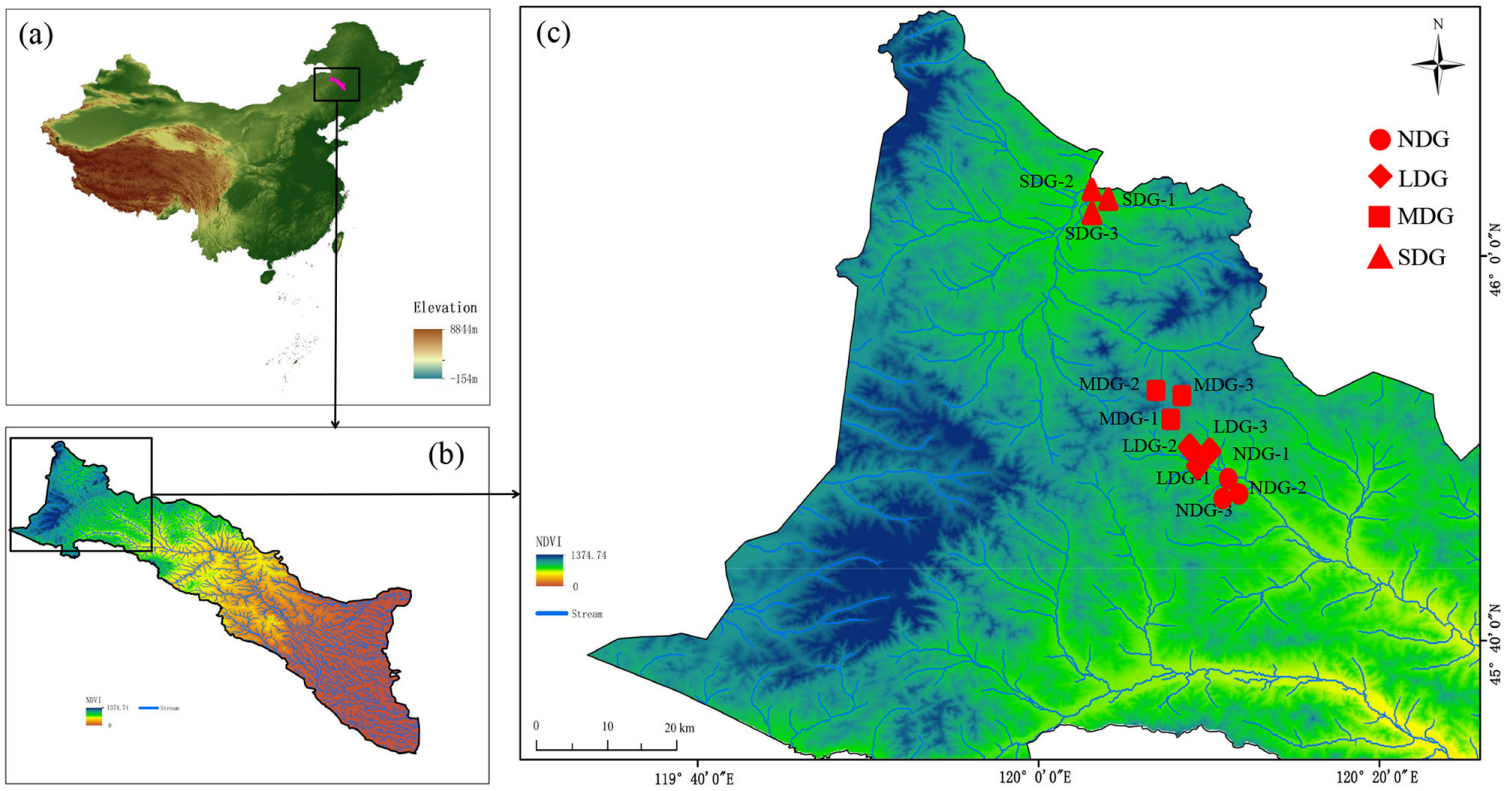
The location of the study area and the sampling sites of southern Greater Khingan; **(a)** Location of southern the Greater Khingan (the map was colored by elevation). **(b)** The distribution of the normalized difference vegetation index (NDVI) across the watershed (the map was colored by NDVI). **(c)** The distribution of the 12 sampling sites (the map was colored by NDVI). NDG, non-degraded grasslands; LDG, lightly degraded grasslands; MDG, moderately degraded grasslands; SDG, severely degraded grasslands.

Based on the combined assessment of plant coverage, vegetation, and soil fertility, four types of grasslands with different degrees of degradation were randomly selected: non-degraded grasslands (NDG), lightly degraded grasslands (LDG), moderately degraded grasslands (MDG), and severely degraded grasslands (SDG) (Li et al., 2023). Each degraded grassland type was randomly set up in three replicates (100 × 100 m^2^ each) with a > 50 m distance between each plot (Figure 1, Supplementary Figure S1), which surpassed the space pertinence of microbial variables (Franklin and Mills, 2003). The detailed information on the sample plots is shown in Supplementary Table S1. Five soil samples from each plot (0–10 cm in depth) were randomly collected using a soil auger (5 cm inner diameter) and composited. The auger was washed with sterile water and air-dried before each sampling. The composite samples were sieved separately through a 2 mm sieve to remove root or other plant materials, loaded into a sterilized self-sealing bag at low temperature, and quickly brought back to the laboratory. Soil samples were divided into two parts: one part was used for analysis of soil properties and the other was stored in a −80°C freezer, which was used for analysis of soil microbial communities.

### Analysis of soil characteristics

Soil pH (soil/H_2_O ratio = 1:2.5) was determined using a pH meter (TFE20-FiveEasyTMpH, MettlerToledo, Germany) (Jiang et al., 2024). The measurements of the soil chemical index were performed following the methods described by a previous study (Li et al., 2021a). Soil organic carbon (SOC) content was measured by oxidizing with potassium dichromate. Total nitrogen (TN) and available nitrogen (AN) were determined using the Kjeldahl and alkali diffusion methods, respectively. Total phosphorus (TP) content was determined using the spectrophotometric ammonium molybdate-ascorbic acid method. Available phosphorus (AP) content was determined using the molybdenum blue method (Xiong et al., 2015). Total potassium (TK) and soil available potassium (AK) contents were measured using the flame emission technique with an FP6431 flame photometer (Shanghai, China).

Soil enzyme activity was measured using a soil enzyme kit (Nanjing Jiceng Biotechnology Co., Ltd., Nanjing, Jiangsu, China) and an applicable UV-Vis spectrometer (UV-8000, Yuan Analysis Instrument Co., Ltd., Shanghai, China). All enzyme activities were determined by air-drying mass. The soil enzyme activity indicators and their notations were as follows: urease (UE), dehydrogenase (DHA), sucrase (SC), cellulase (CL), β-glucosidase (β-GC), alcalase protease (ALPT), and catalase (CAT).

Total concentration of trace elements, iron (Fe), manganese (Mn), nickel (Ni), copper (Cu), zinc (Zn), chromium (Cr), arsenic (As), cadmium (Cd), and lead (Pb) were determined after digestion with HF-HNO_3_-H_2_O_2_ (1:2.5:1, v/v/v) at 160°C for 9 h by using a electrothermal constant temperature blast drying (Wang et al., 2008a).

### Soil DNA extraction, pcr amplification, and illumina miseq sequencing

Total microbial genomic DNA was extracted from 0.5 g soil samples using the E.Z.N.A. Soil DNA Kit (Omega Bio-tek, Norcross, GA, U.S.). The DNA extract was verified using 1% agarose gel, and DNA concentration and quality were evaluated by a NanoDrop 2000 UV–vis spectrophotometer (Thermo Scientific, Wilmington, USA). The V3-V4 region of the bacterial 16S rRNA gene was amplified with the primers 338F (5′-ACTCCTACGGGAGCAGCAG−3) and 806R (5′-GGAC TACHGGGTWTCTAAT−3′) (Liu et al., 2016). For fungal, PCR reactions of the primers ITS1F (5′-CTTGGTCATTTAGAGGAAGTAA−3′) and ITS2R (5′-GCTGCGTTCTTCATCGATGC−3′) were utilized to amplify the ITS1 region (Chen et al., 2023). PCR analysis was performed in triplicate with the following thermal program: 10 ng of genomic DNA, 5 μL of reaction buffer, 4 μL of GC buffer, 2 μL of dNTP, 0.8 μL of each primer, 0.25 μL of Q5 DNA polymerase (New England Biolabs, USA), and supplemented the total system with sterilized ultrapure water to 20 μL. The PCR amplification cycling conditions were as follows: initial denaturation at 95°C for 3 min, followed by 27 cycles of denaturing at 95°C for 30 s, annealing at 55°C for 30 s and extension at 72°C for 45 s, and single extension at 72°C for 10 min, and end at 4°C. The PCR products were further extracted in a 2% agarose gel and purified using the AxyPrep DNA Gel Extraction Kit (Axygen Biosciences, USA) as per the manufacturer's instructions. The purified amplicants were then quantified using the Quantus™ Fluorometer (Promega, USA). Finally, the purified amplicants were pooled equally and subjected to paired-end sequences (2 × 300) on an Illumina MiSeq platform (Illumina, San Diego, USA) following the standard protocols described by Majorbio Bio-Pharm Technology Co. Ltd. (Shanghai, China).

### Processing of sequencing data

A fastp (https://github.com/OpenGene/fastp, version 0.19.6) software was used to control the quality of the double-ended original sequencing sequence (Cheng et al., 2018), The FLASH (http://www.cbcb.umd.edu/software/flash, version 1.2.11) software was used for splicing (Magoc and Salzberg, 2011), and the DADA2 plug-in in the Qiime2 process was used to reduce the noise of the optimized sequence after quality control splicing (Callahan et al., 2016). In order to minimize the impact of sequencing depth on the subsequent analysis of Alpha and Beta diversity data, the number of all sample sequences was flattened to 20,000. After flattening, the average sequence coverage of each sample was still 99.09 %. Based on the silva138/16s_bacteria and unite8.0/its_fungal database (v 138), the Naive Bayes classifier in Qiime2 was used to perform taxonomic analysis of ASVs.

### Statistical analyses

One-way analysis of variance (ANOVA) of soil properties, enzyme activity, soil metal elements, and soil microbial community was performed using the generalized linear model (GLM) in SAS 9.3 software (SAS Institute, Inc., Cary, NC, USA). Significance was calculated using Duncan's test (*P* < 0.05). Species diversity (Shannon-Wiener and Simpson) and richness (Chao1 and ACE) indices were used to assess bacterial and fungal alpha diversity. The relationships among the bacterial and fungal communities were analyzed using principal coordinate analysis (PCoA) in the statistical software R (Ren et al., 2022). PICRUST 2 (http://huttenhower.sph.harvard.edu/galaxy) and FUNGuild (http://www.funguild.org/) were used to analyze and predict the bacterial and fungal community functions, respectively. To improve the normality and reduce the non-linearity of multivariate statistical analysis, function vif.cca was implemented in the vegan package in R, and the environmental variables (soil properties, enzyme activity, and soil metal elements) were log-transformed [log (x+1)] except pH. The variance inflation factor (VIF) for each environmental variable was calculated. The VIF values for pH, SOC, TN, AN, DHA, β-GC, Fe, Ni, Cu, Zn, Cr, Ca, As, and Cd were higher than 10 and were removed. Redundancy analysis (RDA) was used to analyze the relationships between soil microbial community composition and environmental factors. The resulting model was tested using Monte Carlo permutation with 999 iterations (Li et al., 2021a). In addition, “vegan” and “ggcor_master” were used to conduct Random Forest and Mantel tests to identify significant environmental variables influencing the composition and dominance of microbial communities.

## Results

### Effects of degraded grassland on soil properties

**Figure 2:**
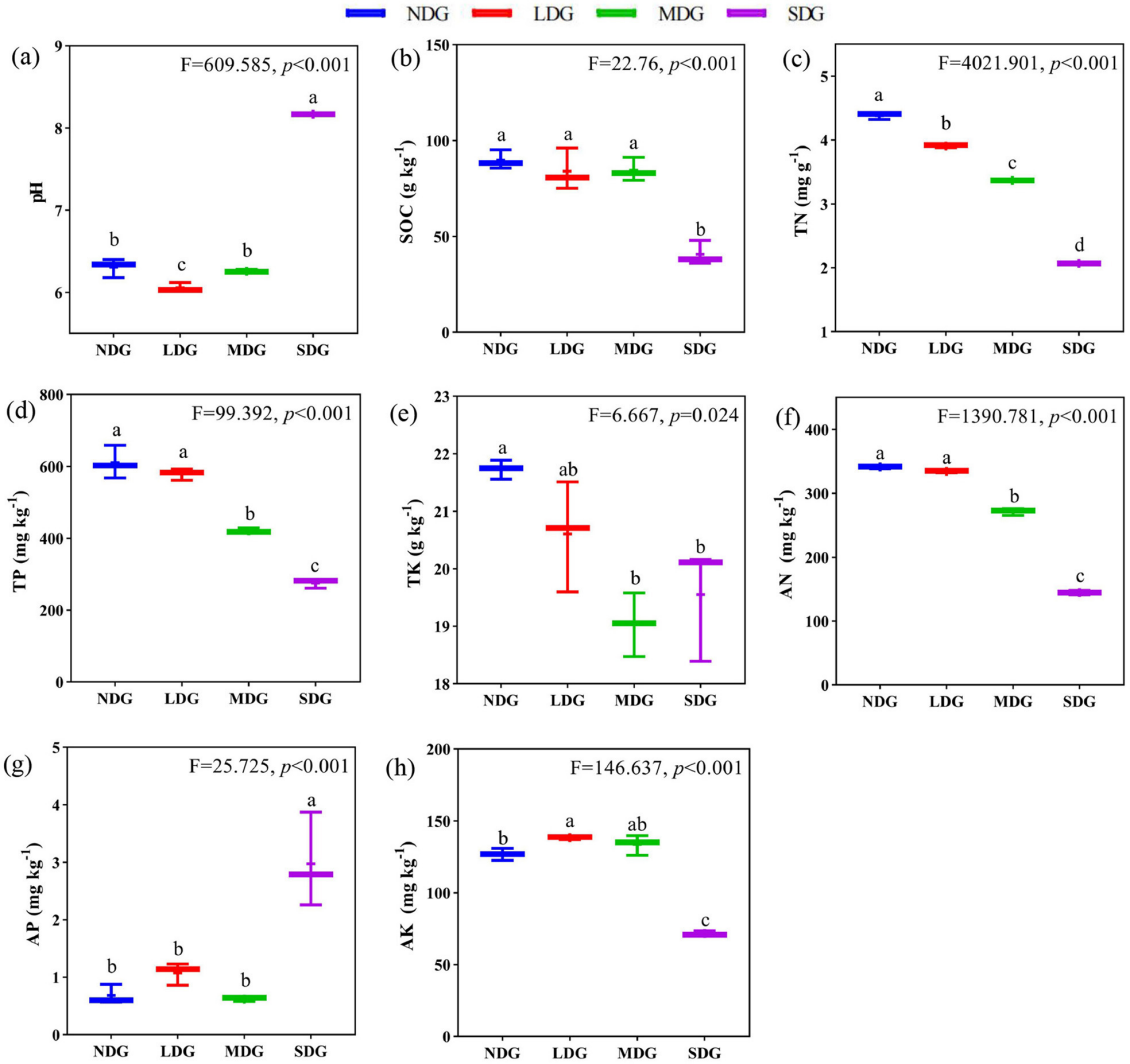
Effects of grassland degradation gradient on soil properties. Different letters **(a–h)** indicate significant differences (*P* < 0.05) among grassland degradation gradient. NDG, non-degraded grasslands; LDG, lightly degraded grasslands; MDG, moderately degraded grasslands; SDG, severely degraded grasslands; SOC, soil organic carbon; TN, total nitrogen; TP, total phosphorus; TK, total potassium; AN, available nitrogen; AP, available phosphorus; AK, available potassium.

With increased grassland degradation, SOC, TN, TP, TK, AN, and AK contents decreased significantly (Figures 2b,c,d,e,f,h) (*P* < 0.05). Compared with NDG, SOC was not significantly different in LDG and MDG (*P* > 0.05). However, it decreased significantly (by 54.67 %) in SDG. The soil TN, TP, TK, AN, and AK were significantly decreased by 52.74 %, 54.7%, 10.03%, 57.63%, and 43.41%, respectively, in SDG compared with NDG. While pH and AP were significantly increased with the grassland degradation (*P* < 0.01), these variables increased by 22.8 % and 77.1 %, respectively, in SDG compared with NDG (Figures 2a,g).

### Effects of degraded grassland on soil enzyme activity

**Figure 3:**
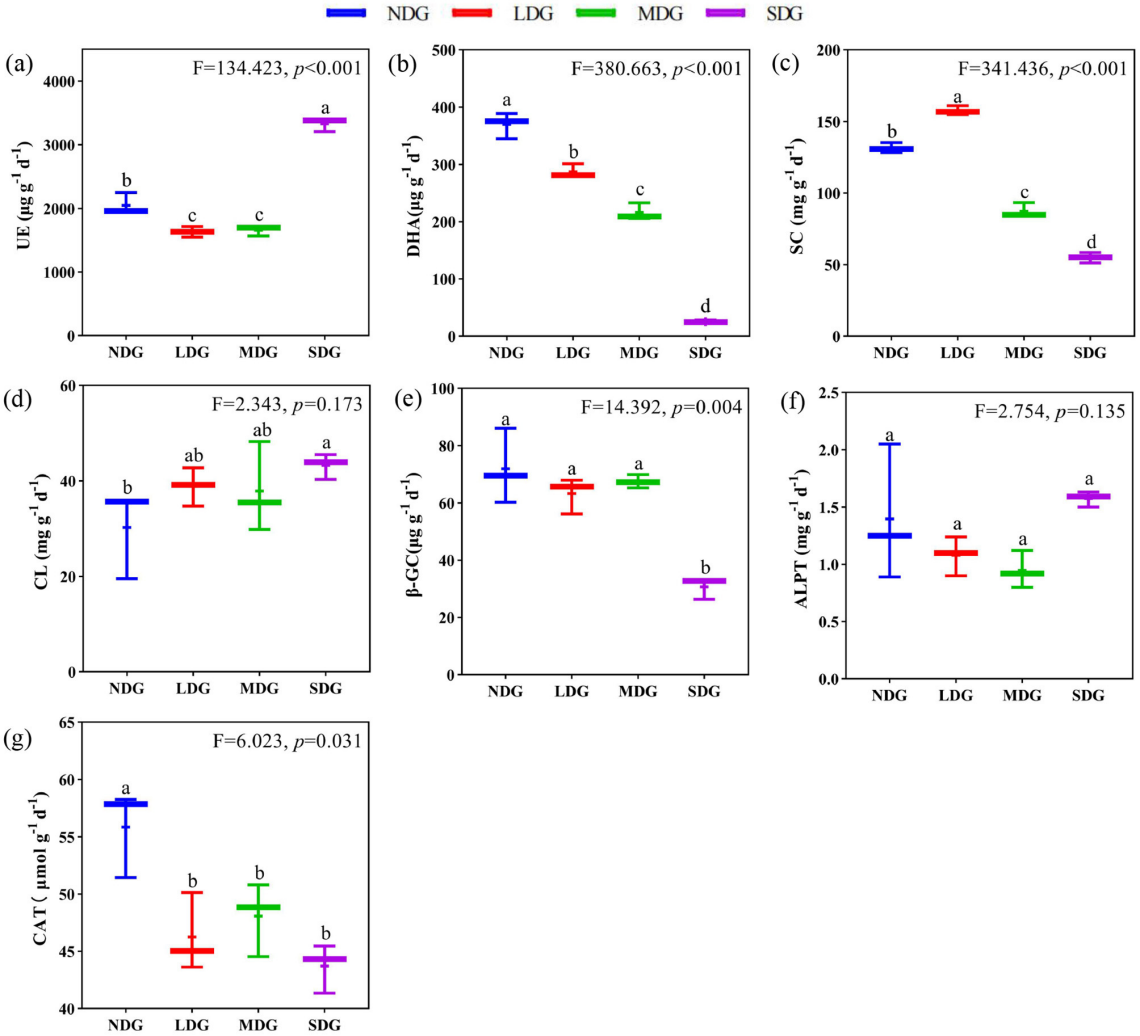
Effects of grassland degradation gradient on soil enzyme activity. Different letters **(a–g)** indicate significant differences (*P* < 0.05) among grassland degradation gradient. NDG, non-degraded grasslands; LDG, lightly degraded grasslands; MDG, moderately degraded grasslands; SDG, severely degraded grasslands; UE, urease; DHA, dehydrogenase; SC, sucrase; CL, cellulase; β-GC, β-glucosidase; ALPT, alkaline phosphatase; CAT, catalase.

The soil enzyme activity significantly differed across the grassland degradation gradient (Figures 3a–g). Soil DHA, SC, β-GC, and CAT contents decreased significantly (*P* < 0.05) with increasing grassland degradation. compared with NDG, DHA, SC, CAT, and β-GC in SDG were reduced by 93.03%, 58.18%, 21.73%, and 57.34%, respectively. However, UE showed a significant decreasing and then increasing trend across the grassland degradation gradient (*P* < 0.01), with a significant increase in SDG by 50.91% compared with LDG. Also, CL increased significantly by 42.87% in SDG compared with NDG. In contrast, there was no significant difference in ALPT across the grassland degradation gradient (*P* > 0.05).

### Effects of degraded grassland on soil metal elements

**Figure 4:**
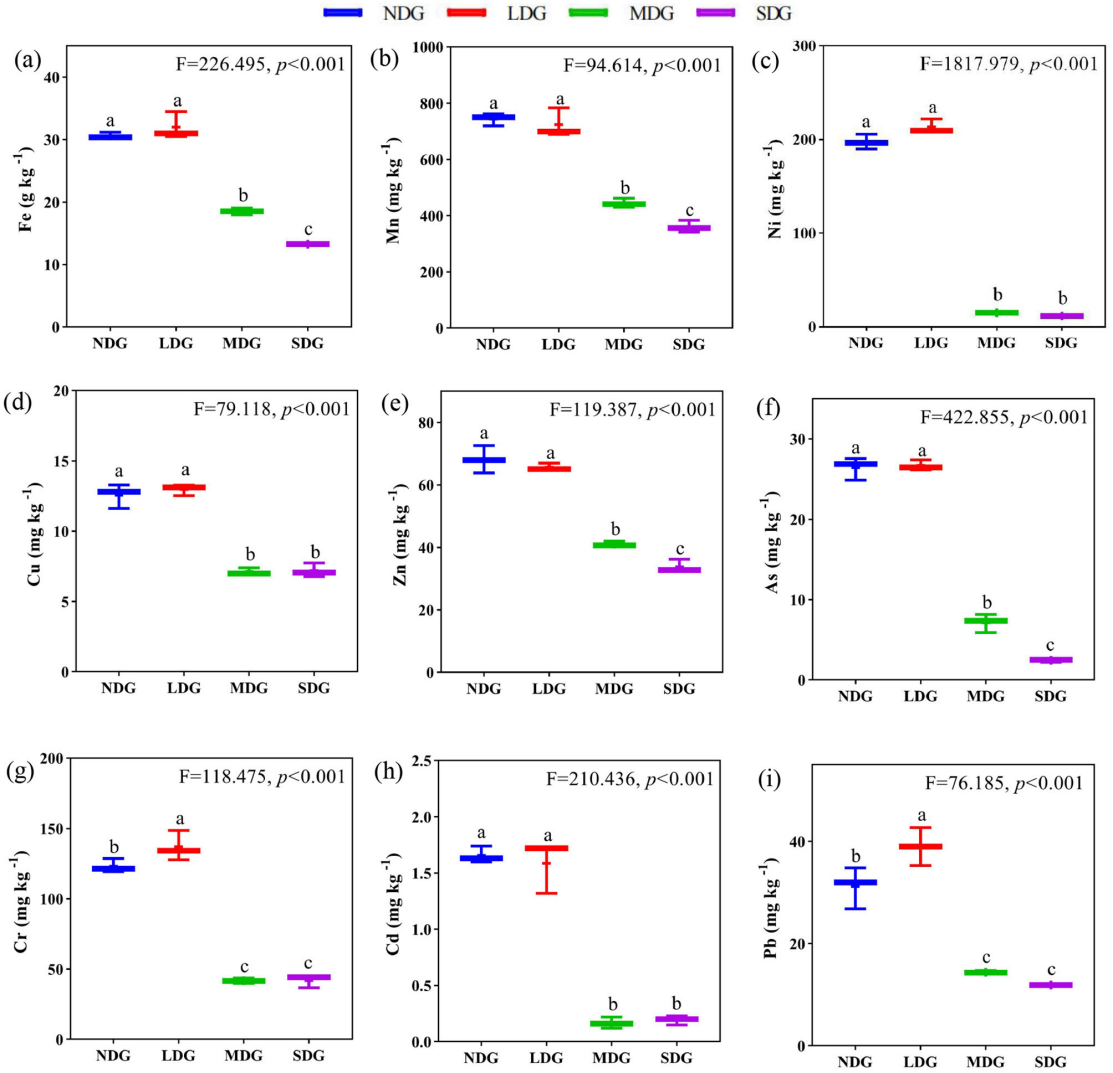
Effects of grassland degradation gradient on soil metals elements. Different letters **(a–i)** indicate significant differences (*P* < 0.05) among grassland degradation gradient. NDG, non-degraded grasslands; LDG, lightly degraded grasslands; MDG, moderately degraded grasslands; SDG, severely degraded grasslands.

The soil metal elements significantly differed across the grassland degradation gradient (Figures 4a–i). With increasing grassland degradation, soil Fe, Mn, Ni, Cu, and Zn contents decreased significantly (*P* < 0.01). compared with NDG, soil Fe, Mn, Ni, Cu, and Zn contents decreased significantly by 56.65%, 51.52%, 94.12%, 42.74%, and 50.48%, respectively, in SDG. However, Cr, Cd, and Pb showed increasing and decreasing trend along the grassland degradation gradient (*P* < 0.01), with the elements decreased significantly by 69.39%, 90.86%, and 69.44%, respectively, in SDG compared with LDG.

### Effects of degraded grassland on soil microbial communities

**Figure 5:**
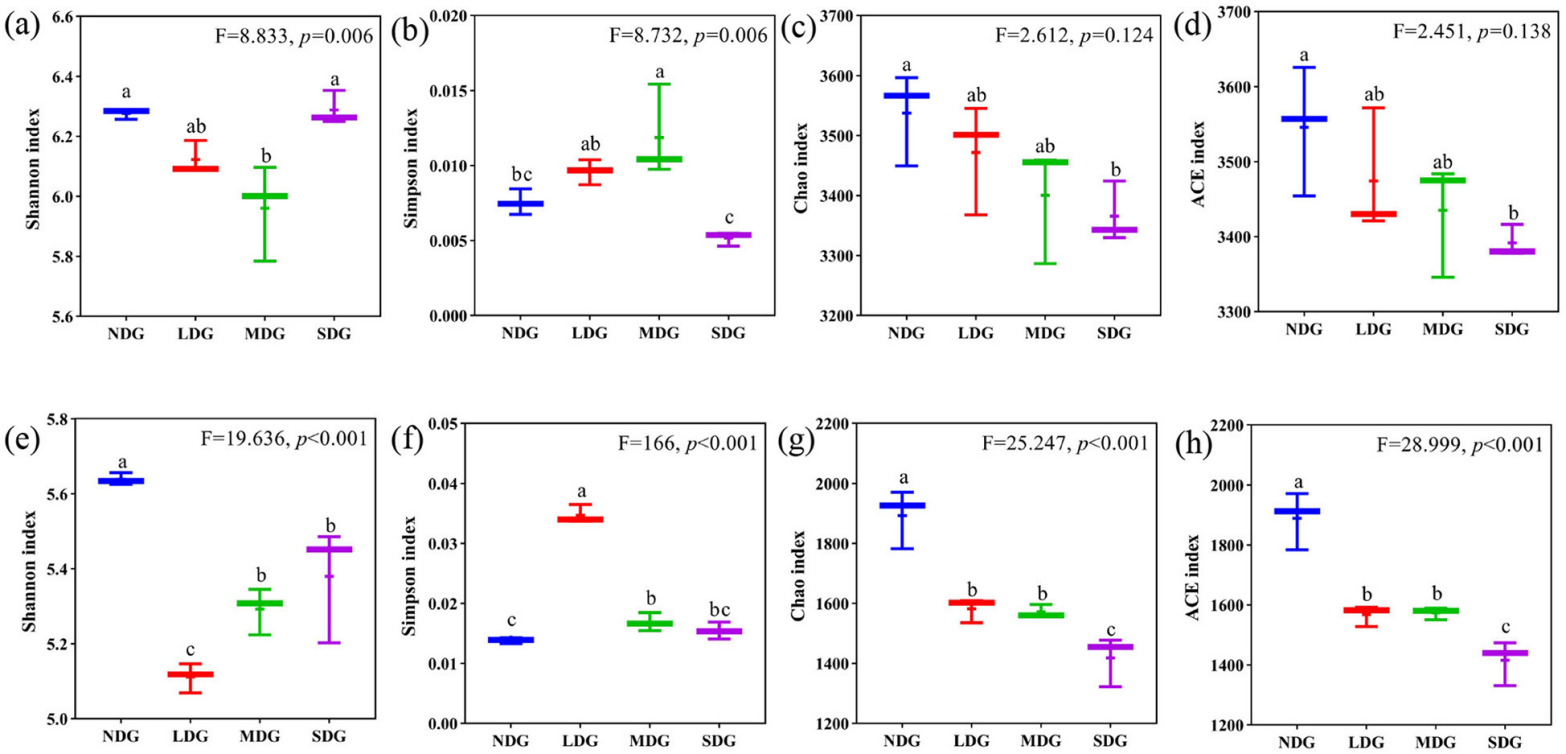
Effects of grassland degradation gradient on diversity of the microbial community. Different letters indicate significant differences (*P* < 0.05) among grassland degradation gradient. **(a–d)** were Shannon, Simpson, Chao1, and ACE indexes of bacterial communities, respectively; **(e–h)** were Shannon, Simpson, Chao1, and ACE indexes of fungal communities, respectively. NDG, non-degraded grasslands; LDG, lightly degraded grasslands; MDG, moderately degraded grasslands; SDG, severely degraded grasslands.

**Figure 6:**
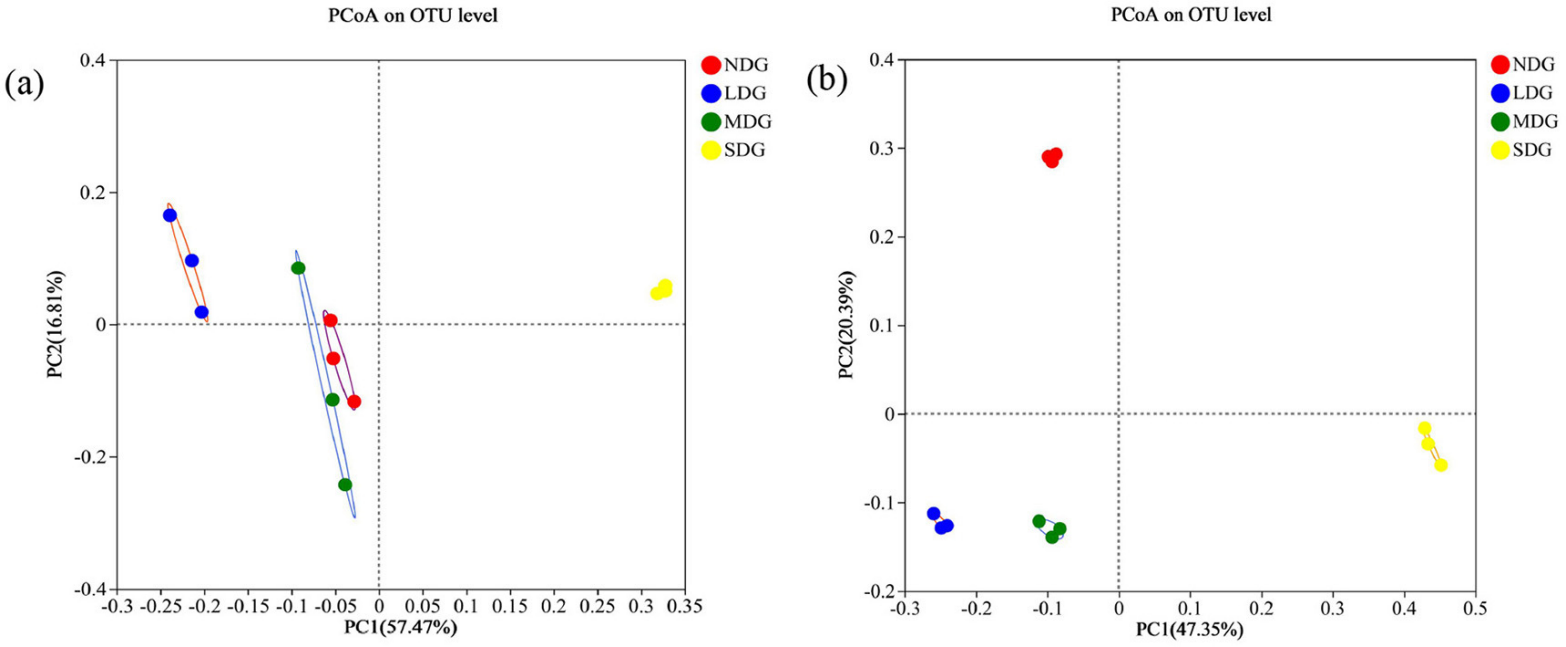
PCoA analysis of soil bacterial **(a)** and fungal **(b)** communities in grassland degradation gradient. NDG, non-degraded grasslands; LDG, lightly degraded grasslands; MDG, moderately degraded grasslands; SDG, severely degraded grasslands.

The Shannon, Simpson, Chao1, and ACE indices were employed to analyze and evaluate the richness and diversity of microbial communities in the different degraded grasslands. For the bacterial community, Shannon index was highest in SDG and lowest in MDG, while Simpson index was highest in MDG and lowest in SDG. With respect to the richness indices, Chao1 and ACE were the highest in NDG and the lowest in SDG (Figures 5a–d). For the fungal community, Shannon index was the highest in NDG and the lowest in LDG, while Simpson index was the highest in LDG and the lowest in NDG. However, Chao1 and ACE were significantly lower in SDG compared with the other degraded grasslands (Figures 5e–h) (*P* < 0.01). Principal coordinate analysis (PCoA) based on Bray-Curtis showed the compositional differences in bacterial and fungal communities across the grassland degradation gradient, with both communities separated into four distinct groups (Figures 6a,b).

### Effects of degraded grassland on soil microbial community composition

**Figure 7:**
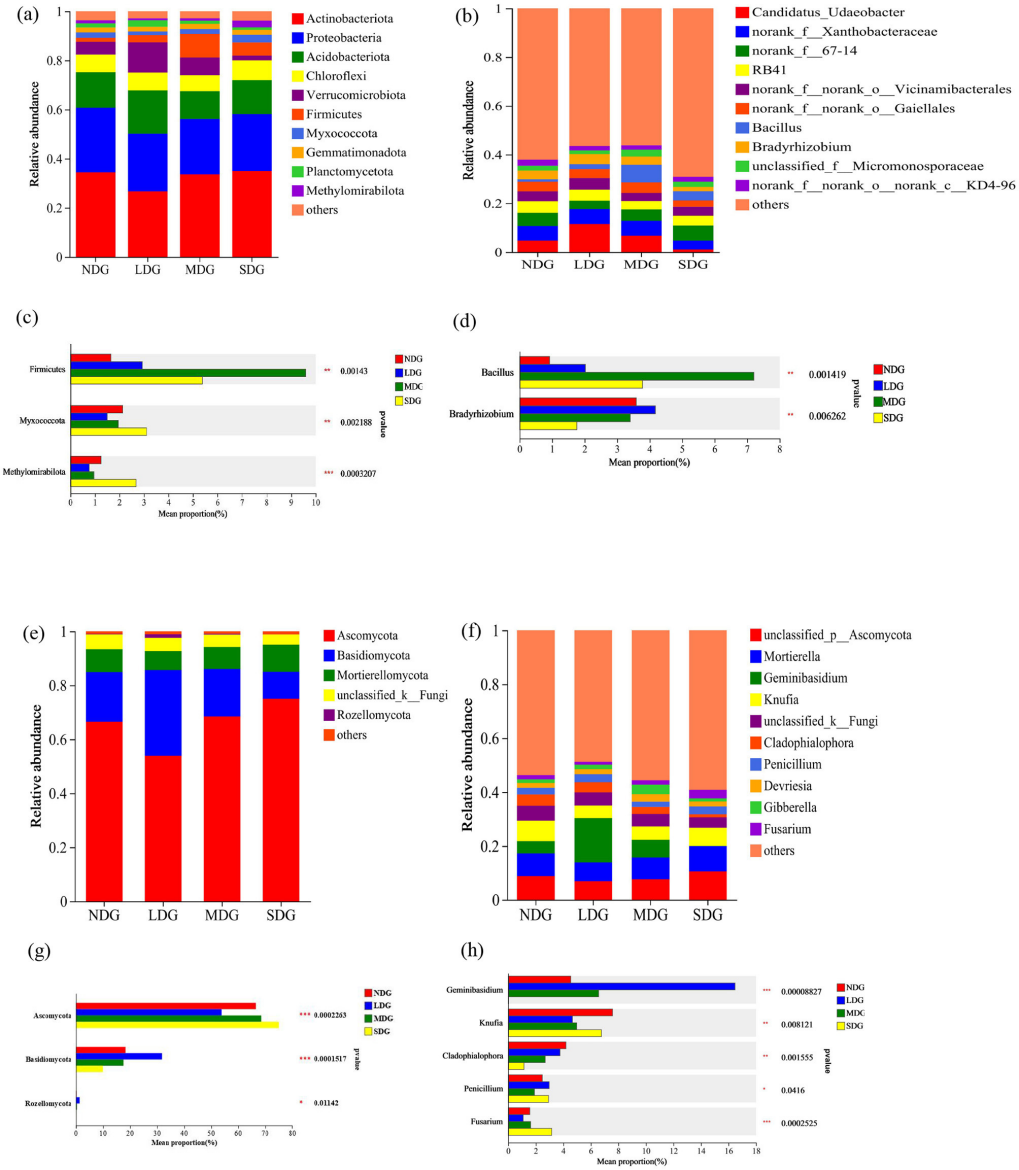
Effects of grassland degradation gradient on soil bacterial phyla **(a)** and genus **(b)** and fungal phyla **(e)** and genus **(f)** community composition. One-way analysis of variance bar plots of the bacterial phyla **(c)** and genus **(d)** and fungal phyla **(g)** and genus **(h)**. *: *P* < 0.05, **: *P* < 0.01, ***: *P* < 0.001. NDG, non-degraded grasslands; LDG, lightly degraded grasslands; MDG, moderately degraded grasslands; SDG, severely degraded grasslands.

At the phylum level, the soil bacterial community mostly consisted of Actinobacteria, Proteobacteria, Acidobacteria, Chloroflexi, Verrucomicrobiota, and Firmicutes (Figure 7a). Among the phyla, the abundance of Actinobacteria, Proteobacteria, and Myxococcota tended to decrease and then increase with the increase of grassland degradation. The relative abundance of Verrucomicrobiota and Firmicutes, however, tended to increase initially and then decrease across the degradation gradient. The relative abundance of Myxococcota (3.11%) and Methylomirabilota (2.72%) in SDG was significantly higher than the other degradation levels (*P* < 0.05) (Figure 7c). For the fungal community, the dominant phyla across all degradation levels were comparable (Figure 7e). The abundance of Ascomycota initially decreased and then increased with the increase in degradation, and its average abundance in NDG, LDG, MDG, and SDG was 66.54%, 53.91%, 68.48%, and 75.05%, respectively (*P* < 0.05) (Figure 7g). With the increase in grassland degradation, the relative abundance of Basidiomycota and Rozellomycota increased initially and then decreased. However, the relative abundance of unclassified _k_ fungal phyla decreased gradually from low to high degradation. At the genus level, there was a significant difference in the relative abundance of bacterial and fungal communities across different degraded levels (Figures 7b, d, f, h). The relative abundance of *Bradyrhizobium, Geminibasidium*, and *Cladophialophora* reduced significantly in SDG (*P* < 0.05).

### Prediction of soil microbial functional characteristics across the grassland degradation gradient

**Figure 8:**
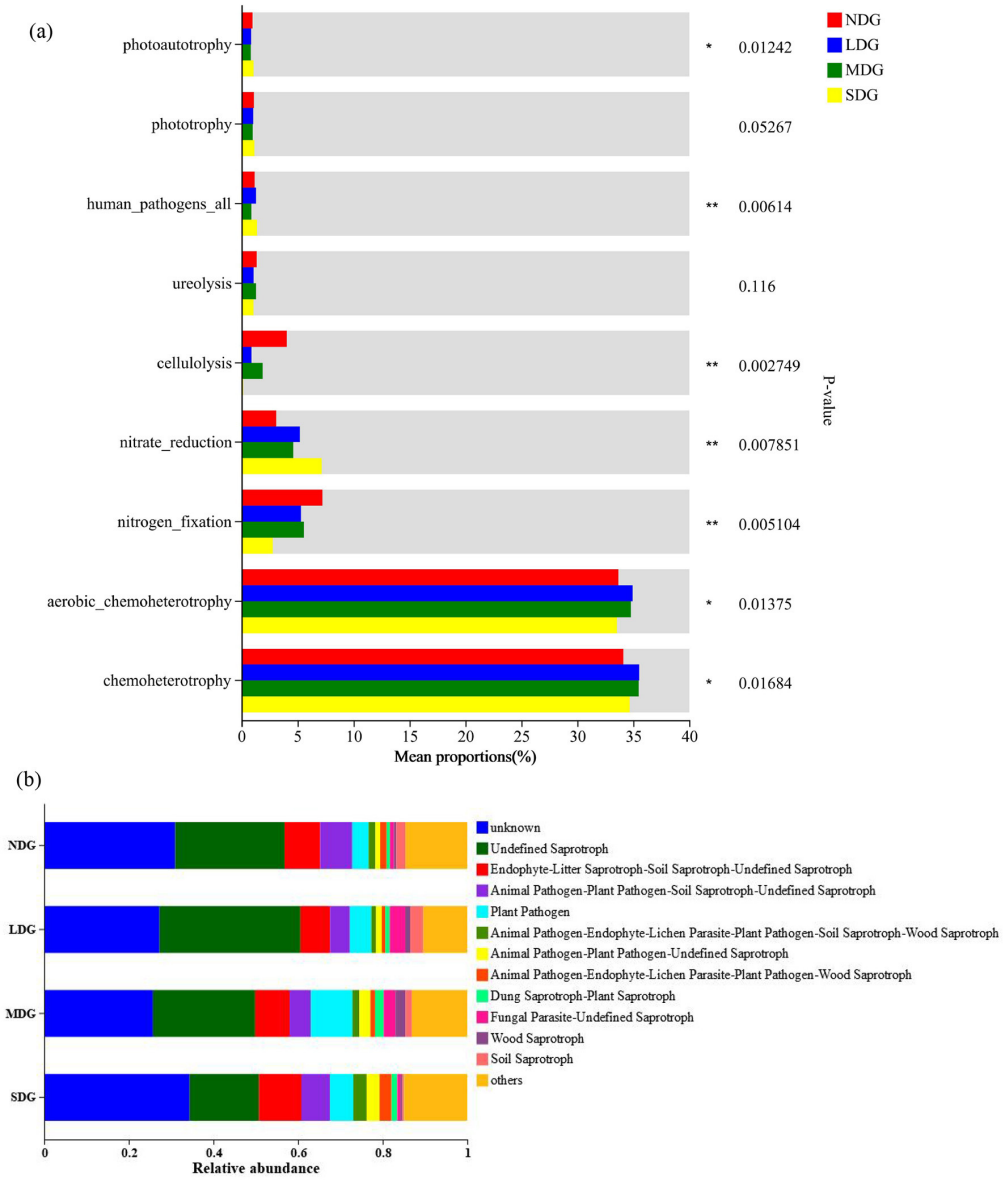
Predicted functional profiles of the soil bacteria **(a)** and fungal **(b)** in the grassland degradation gradient. *: *P* < 0.05, **: *P* < 0.01; NDG, non-degraded grasslands; LDG, lightly degraded grasslands; MDG, moderately degraded grasslands; SDG, severely degraded grasslands.

The effects of metabolic pathways on microbial communities were quantified by predicting the functional characteristics of microbial communities. The bacterial OTUs were classified into 9 functional guilds (Figure 8). With the increase of grassland degradation, bacterial functional groups related to nitrogen fixation and cellulolysis were significantly decreased. In contrast, those related to nitrate reduction and human_pathogens_all increased with grassland degradation (Figure 8a). For fungal functional prediction, the relative abundance of “Endophyte-Litter Saprotroph-Soil Saprotroph-Undefined Saprotroph,” “Animal Pathogen-Plant Pathogen-Undefined Saprotroph,” “Animal Pathogen-Endophyte-Lichen Parasite-Plant Pathogen-Soil Saprotroph-Wood Saprotroph,” and “Animal Pathogen-Plant Pathogen-Soil Saprotroph-Undefined Saprotroph” increased significantly as the grassland degradation increased, while “Undefined Saprotroph,” “fungal Parasite-Undefined Saprotroph,” and “Soil Saprotroph” decreased with increasing severity of degradation (Figure 8b).

### Relationship between soil microbial communities and soil environmental factors

**Figure 9:**
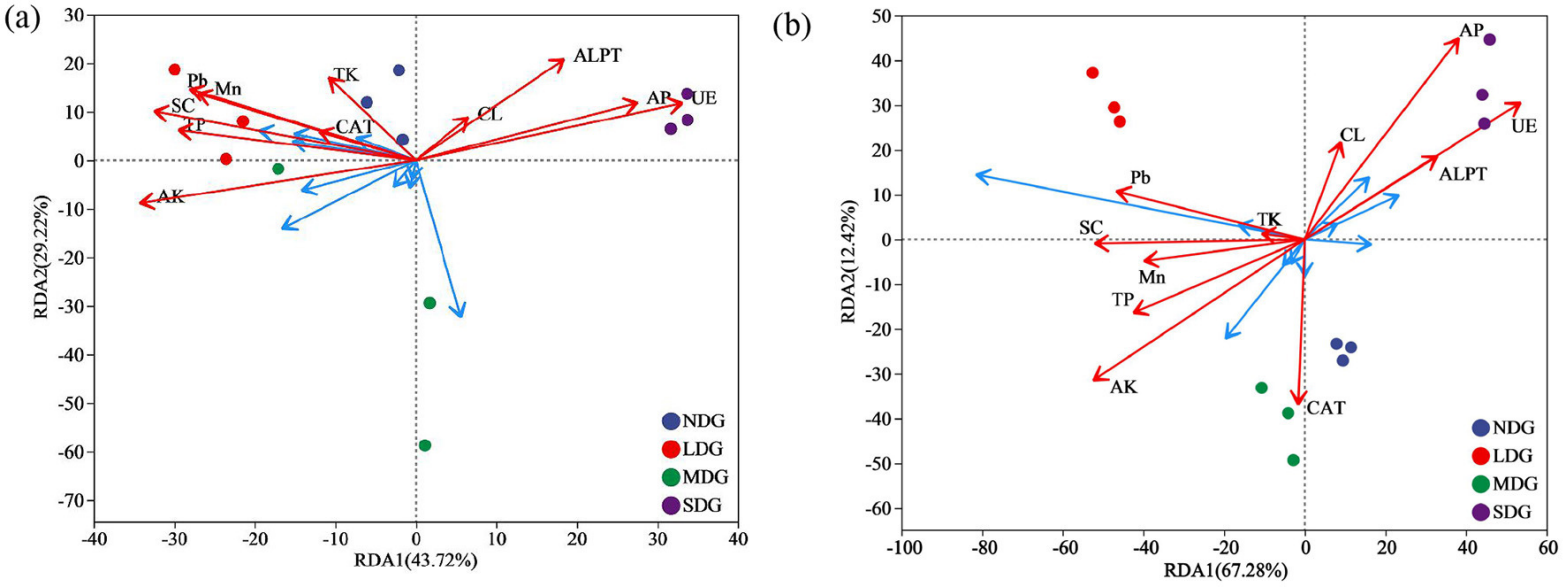
The redundancy analysis (RDA) of the bacterial **(a)** and fungal **(b)** communities with soil environmental factors in soil with grassland degradation gradient. NDG, non-degraded grasslands; LDG, lightly degraded grasslands; MDG, moderately degraded grasslands; SDG, severely degraded grasslands. TP, total phosphorus; TK, total potassium; AP, available phosphorus; AK, available potassium; UE, urease; SC, sucrase; CL, cellulase; ALPT, alkaline phosphatase; CAT, catalase.

**Figure 10:**
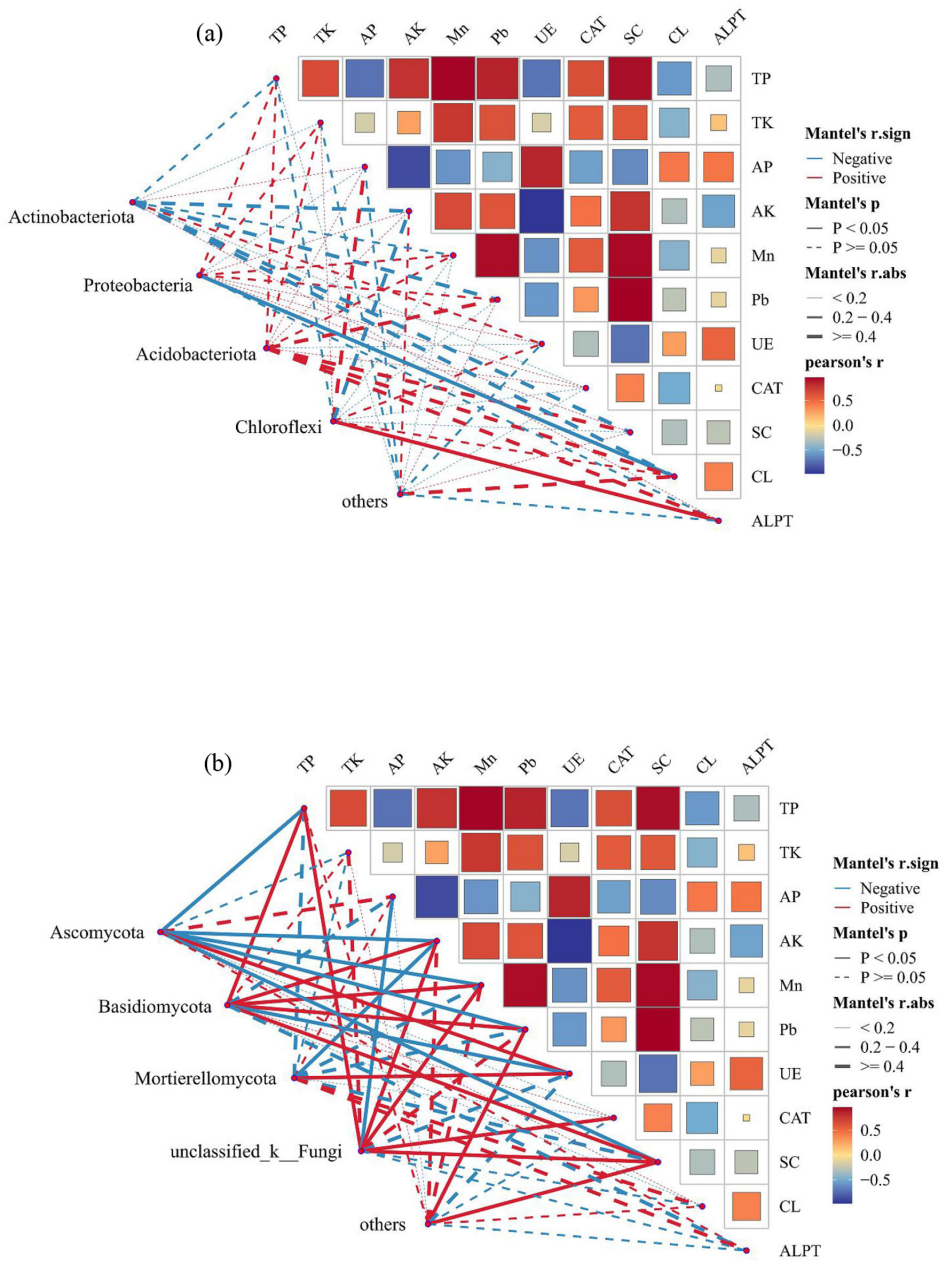
Partial Mantel test examined the relationship between dominant phyla of soil bacteria **(a)** and fungal **(b)** (relative abundance > 5%) and soil environmental factors. The edge width corresponds to the Mantel's r statistic for the corresponding distance correlation, the edge color indicates positive and negative correlation, and the edge shape indicates statistical significance.

The RDA and Monte Carlo permutation test identified TP, AP, AK, Mn, Pb, UE, SC, and ALPT as important factors driving soil bacterial community (Figure 9a; Table 1). However, the soil fungal community was mainly affected by TP, AP, AK, Pb, UE, and SC (Figure 9b). The bacterial community was dominated by Actinobacteriota, Proteobacteria, Acidobacteriota, and Chloroflexi, and fungal community was dominated by Ascomycota, Basidiomycota, and Mortievellomycota. We also analyzed the correlation between species abundance and soil environmental factors. Proteobacteria showed a significant negative correlation with CL (*P* < 0.05). Chloroflexi showed significant positive association with ALPT (*P* < 0.05). However, Actinobacteriota and Acidobacteriota had no significant relation with soil physicochemical properties (*P* > 0.05) (Figure 10a). In terms of fungal, Ascomycota showed a significant negative correlation with TP, AK, Mn, Pb, and SC (*P* < 0.05) and a positive association with UE (*P* < 0.05). In contrast, Basidiomycota showed a significant positive correlation with TP, Pb, and SC (*P* < 0.05) and a negative association with UE (*P* < 0.05). Mortievellomycota showed a significant positive correlation with UE (*P* < 0.05) and a negative correlation with AK (*P* < 0.05) (Figure 10b).

**Table 1:** Monte Carlo permutation test of environmental factors and microbial communities.

**Parameter**	**Bacteria**				**Fungal**			
	**RDA1**	**RDA2**	**R** ^2^	***P***	**RDA1**	**RDA2**	**R** ^2^	***P***
TP	−0.9786	0.2056	0.6757	**0.01**	−0.9328	−0.3605	0.5239	**0.041**
TK	−0.5363	0.844	0.2899	0.225	−0.993	0.118	0.0246	0.895
AP	0.9194	0.3934	0.6664	**0.015**	0.6479	0.7617	0.8899	**0.001**
AK	−0.9682	−0.2503	0.9357	**0.001**	−0.8571	−0.5151	0.9578	**0.005**
Mn	−0.8922	0.4516	0.6839	**0.009**	−0.9927	−0.1208	0.4035	0.122
Pb	−0.8865	0.4627	0.7445	**0.005**	−0.9745	0.2246	0.5816	**0.026**
UE	0.9425	0.3341	0.9211	**0.001**	0.8685	0.4956	0.972	**0.002**
CAT	−0.8997	0.4365	0.1263	0.577	−0.0436	−0.999	0.3423	0.183
SC	−0.955	0.2968	0.8643	**0.001**	−0.9998	−0.0181	0.6899	**0.007**
CL	0.5951	0.8036	0.0809	0.693	0.3817	0.9243	0.134	0.551
ALPT	0.6632	0.7484	0.5666	**0.021**	0.8707	0.4917	0.3578	0.109

TP, total phosphorus; TK, total potassium; AP, available phosphorus; AK, available potassium; UE, urease; SC, sucrase; CL, cellulase; ALPT, alkaline phosphatase; CAT, catalase. *P* values based on 999 permutations.

## Discussion

### Soil properties, enzyme activity, and soil metal elements in response to grassland degradation

Grassland degradation is a complex ecological process (Zhang et al., 2024), characterized primarily by vegetation damage and soil degradation (Chen et al., 2014). Soil provides substrates and environment for plant growth, with other important ecosystem functions, including nutrient cycling and supporting aboveground and belowground diversity (Du et al., 2019). Soil TN and SOC are considered important indicators of grassland quality (Ma et al., 2023). In our study, the lowest TN and SOC contents was reported from the SDG, which are potentially due to the alteration in litter accumulation and subsequent carbon input to the soil (Han et al., 2020). The AP increased with the intensity of grassland degradation, which may be due to a decrease in organic matter replenishment to the soil by plant residues, which reduces phosphorus fixation and increases AP content. Soil enzymes are one of the most active organic components in the soil and are involved in various soil biochemical processes, assisting plant growth (Li et al., 2014). Lin et al. (2017) showed that UE activity increased with increasing grazing intensity, due to the increase in fecal input from livestock under heavy grazing, which resultantly increase soil organic nitrogen content. Our study found that soil UE activity increases gradually with the increase in grassland degradation, the organic nitrogen content of the soil increased due to the influence of the excreta of burrowing animals such as prairie pika and zokors, which led to the elevation of UE activity. In addition, we found that except for soil pH, AP, UE, and CL, soil AN, AK, TN, TP, TK, and enzyme activity (DHA, CAT, β-GC, SC) significantly differed, which is consistent with previous studies (Li et al., 2023; Bai et al., 2023). The main reason for this is that as degradation increases, the distribution of grassland vegetation cover further decreases, leading to less litter input and accumulation. This exposes the soil surface to radiation and heat, resulting in soil erosion from wind and rainfall (Dai et al., 2023). Second, human activities (e.g., grazing and reclamation) lead to a reduction in above- and below-ground biomass, which further leads to a reduction in root exudates and apoplastic material in the soil, weakening the effects of vegetation residues on soil nutrients, enzyme activity, and microbial abundance (Zeng et al., 2017). Additionally, previous studies have shown that increased grassland degradation enhances the enrichment of soil metal elements (e.g., Cd, Pb, and Cr) (Zhou et al., 2020; Jin et al., 2024). In contrast, we found decreased soil metal elements with increased grassland degradation. Our findings may be explained by the location of the study area, which is away from the mining region and is less affected by human activities. In addition, different climatic conditions, vegetation structure, moisture availability, and altitude may have a negative impact on heavy metal content in the soil and reduce their amount (Huo et al., 2022; Mahvi et al., 2022).

### Soil microbial communities in response to grassland degradation

Ecological distribution and variation of microorganisms in grassland soils with different degrees of degradation vary greatly (Reiss et al., 2010). In our study, the Shannon index of bacterial and fungal communities showed a tendency of decreasing and then increasing. However, the Ace index for bacterial and fungal communities gradually decreased as the degradation increased. These results are comparable to the results from a study in degraded alpine meadows (Wang et al., 2023). Variations in microbial communities are associated with the intensities of grassland degradation (Peng et al., 2020b), where the soil environment became harsher, limiting the growth of some microorganisms and reducing the diversity of soil microbial communities. However, the disturbance causes by grassland degradation may create an environment conducive for the growth of some new and more disturbance-tolerant microorganisms (Chen et al., 2011). In addition, we found that a diversity of fungi with different grassland degradation were significantly higher than those of bacteria, which was also revealed by previous studies (Wang et al., 2022; Ma et al., 2023), suggesting that fungal community is more responsive to grassland degradation than the bacterial community. However, Yu et al. (2021) show that soil bacterial community is more sensitive than fungal community in response to degradation in the temperate grassland, which may be because the soil fungal community in temperate grasslands is more resistant to disturbance and tends to have a delayed response (de Vries et al., 2018).

The bacterial communities at the grassland degradation were dominated by Actinobacteria, Proteobacteria, and Acidobacteria in this study, which is consistent with previous studies (Jiang et al., 2024). This is due to the fact that Actinobacteria, Proteobacteria, and Acidobacteria were able to have a higher level of adaptation to degraded grasslands (Zhou et al., 2019). Actinobacteria use spores to resist adverse environmental conditions (Jin et al., 2018). Proteobacterial may play a pivotal role in phylogenetic, ecological and pathogenic values, and participate in energy metabolism, such as the oxidation of organic and inorganic compounds and the acquisition of energy from light (Kumaresan et al., 2018). Acidobacteria is commonly found in nutrition-deficient environments (Thomson et al., 2010). In addition, we found a significant effect of grassland degradation on the relative abundance of Firmicutes, as its relative abundance increased first and then decreased with grassland degradation. This may be due to suitability of the environments for growth at nutrients deficeint conditions. Moreover, the soil of SDG is more barren and limits the availability of organic matter, limiting the growth of Firmicutes (Li et al., 2018). Furthermore, Firmicutes forms spores under harsh environmental conditions (Mukhopadhya et al., 2012).

The dominant groups of soil fungi in differently degraded grassland were Ascomycota and Basidiomycota (Figure 7e), which are common fungal phyla found in degraded grassland due to higher lignin availability in the soil (Han et al., 2024). In our study, the relative abundance of Ascomycota decreased and then increased as the degradation intensified, which is consistent with the results of a previous study (Li et al., 2019). On the one hand, most ascomycetes live on land and their nutritional methods included saprophytic, parasitic, and symbiotic. Saprophytic ascomycetes help break down plant residue and lignin (Jaklitsch et al., 2014). Therefore, the average Ascomycota abundance increased in the severely degraded grasslands, suggesting that saprophytic ascomycetes promote the decomposition of dead roots and animal residues in the degraded grasslands. On the other hand, Ascomycota dominates the environment with high lignin content (Li et al., 2017), with the degradation of grassland led to the reduction of grassland humus, which leading to the relative abundance of Ascomycota fungal decreased, grassland degradation resulted in distribution of *Stellera chamaejasme* in the grassland community enhancement, which in turn affects the production of plant root secretion, resulting in the relative abundance of Ascomycetes fungal increased (Cheng et al., 2021). In addition, our results show that grassland degradation significantly reduces the relative abundance of Basidiomycota, which is due to the fact that Basidiomycota are eutrophic bacteria that prefer a resource-rich, plant-rich environment (Sterkenburg et al., 2015), and perform better in undisturbed environmental (Yu et al., 2021). Different fungal have different preferences for substrate utilization processes, which main reason for the differences between Ascomycetes and Basidiomycota (Li et al., 2019; McGuire et al., 2010).

In our study, we observed the relative abundance of nitrate reduction increases with the degradation of grassland, which due to increased denitrification and result in increased nitrogen loss (Pan et al., 2018). Our results suggest that changes in the putative functionality of soil bacteria with respect to soil N cycling are in agreement with the field measurements by Luo et al. (2020) and Zhou et al. (2019), indicating that grassland degradation has a dramatic impact on the nitrogen and its functions. Extensive studies have shown that the abundance of pathotrophs and saprotrophs were significantly decreased by grassland degradation which may be ascribed to the reduced plant species diversity during degradation (Yu et al., 2021). The fungal guilds examined by the FUNGuild pipeline have shown interesting outcome that the relative abundance of pathotrophs increased while saprotrophs decreased with increasing severity of degradation. This is due to the fact plant pathogens typically feed on plant roots (Che et al., 2019), grassland degradation resulted in distribution of *Stellera chamaejasme* in the grassland community enhancement, which resulting in an increase in the relative abundance of plant pathotrophs. Saprophytic fungi play a key role in the decomposition of litter, due to the reduction of plant litter in the degraded grassland, which decreases plant saprotrophs (Yu et al., 2021).

### Relationship among microbial communities and soil environmental factors

The RDA analysis showed that the environmental factors affected the soil bacterial and fungal communities, which was consistent with the finding of Cheng et al. (2022) that microbial communities with higher relative abundance are significantly affected by environmental factors, but the dominant influencing factors would be different. Bai et al. (2023) show that EC, pH, and AP were the main drivers of soil bacterial community composition, while EC, pH, and SOC were the main drivers of soil fungal community composition. In this study, the results of Mantel tests showed soil TP, AP, AK, Mn, Pb, UE, SC, and ALPT were the main drivers of soil bacterial community composition, while TP, AP, AK, Pb, UE, and SC were the main drivers of soil fungal community composition (Figure 10, Table 1). Huang et al. (2016) reported that TP had an indirect effect on microorganisms mainly by affecting soil carbon cycle and chemical properties, such as pH. Many studies have also reported the general influence of TP on the distribution pattern of soil microbes (Liu et al., 2023; Wang et al., 2008b). This study also found that TP significantly affected soil microbial community composition. However, different microbial populations had different responses to TP; for example, the abundance of Ascomycota was negatively correlated with TP, whereas the abundance of Basidiomycota was positively correlated with TP. Many large-scale studies demonstrated the strong effects of TP on microorganisms, whereas other studies suggested that other factors may be more important than TP. Li et al. (2023) found that soil pH is the most important driving factor of microbial communities in the alpine grassland degradation. These results highlight the importance of soil nutrients influencing the changes in microbial community composition in meadow grasslands undergoing severe degradation (Luo et al., 2023; Ren et al., 2021). Soil enzymes are mainly derived from soil microorganisms, plant root exudates and the decomposition of animal and plant residues (Yao et al., 2024). Previous studies have shown that when the TN content in soil is below a certain threshold, soil microorganisms will secrete more UE activity through their own regulation and convert more effective nitrogen for plant uptake and utilization, resulting in an increase in UE activity (Yang et al., 2023), this is consistent with the results of the present study, where heavily degraded grassland had the lowest soil TN content and enhanced UE activity. This study found that Proteobacteria and CL have a negative correlation, which is due to soil CL is associated with soil respiration, can hydrolyze cellulose, promote carbon cycle (Draganova et al., 2018), while Proteobacteria were eutrophic bacteria, many kinds of bacteria are involved in soil nutrient cycling (Masuda et al., 2024). Soil metals elements may help explain the structure and functions soil microbiomes, e.g., Fe and Mn for microbial respiration, Cu and Zn for competencies, and Fe, Mo, and Ni for N fixation, etc. (Feng et al., 2019). We found that Mn had the strong correlations with microbes, followed by Pb. This was partly expected because previous work suggests the role of Mn in many important enzymatic processes in soils and act as reactants for C, N, and S coupled reactions (Dai et al., 2023).

The partial mental test demonstrated a correlation between the main fungal phylum and most soil nutrients and enzymes, indicating that the fungal group in our study is poorly adapted to the harsh environment. Nevertheless, the reduction in vegetation root systems from degradation can affect the types and quantities of root exudates, negatively influencing the health of the fungal community (Li et al., 2019). Therefore, changes in soil fungal community structure can be used to infer changes in soil nutrients, offering a novel avenue for investigating the consequences of grassland degradation. Furthermore, light may affect carbon recycling and influence microbial communities through plant photosynthesis, and microbial communities would be also strongly influenced by temperature (Luo et al., 2023), and there may be interaction effects between the various factors (Yao et al., 2024). Therefore, a wider range of environmental factors should be further investigated in the future to obtain more comprehensive information.

## Conclusions

This study has shed some light on the response of soil chemical properties, enzyme activity, metal elements, and microbial communities to grassland degradation. It has also revealed the relationships between soil microbial community composition and environmental variables across the degraded grasslands south of the Greater Khingan Mountains, China. The diversity of bacteria decreased and the diversity of fungi increased with the increase of grassland degradation. Our findings underscore the significance of TP, AP, AK, Pb, UE, and SC in influencing the changes in soil bacterial and fungal community composition. In addition, the putative functions revealed from soil bacterial profiles, such as nitrification and nitrate reduction, were seemingly altered during degradation, indicating the potential influence of degradation on soil nutrient cycling. These results provide valuable insights into the functions and services of degraded grassland ecosystems south of the Greater Khingan Mountains. Therefore, for the effective management and development of the grassland ecosystems south of the Greater Khingan Mountains, it is suggested to reduce the current unmanaged grazing and land use, which can improve soil fertility and the health of soil microorganisms.

## Data Availability

The data presented in the study are deposited in the National Center for Biotechnology Information repository, accession number PRJNA1111039 (bacteria) and PRJNA1111151 (fungal).

## References

[B1] Bai Z. Y. Jia A. M. Li H. X. Wang M. J. Qu S. M. (2023). Explore the soil factors driving soil microbial community and structure in Songnen alkaline salt degraded grassland. Front. Plant Sci. 14:1110685. 10.3389/fpls.2023.1110685 37229114 PMC10203596

[B2] Bardgett R. D. van der Putten W. H. (2014). Belowground biodiversity and ecosystem functioning. Nature 515, 505–511. 10.1038/nature13855 25428498

[B3] Callahan B. J. McMurdie P. J. Rosen M. J. Han A. W. Johnson A. J. A. Holmes S. P. (2016). DADA2: high-resolution sample inference from Illumina amplicon data. Nat. Methods. 13, 581–583. 10.1038/nmeth.3869 27214047 PMC4927377

[B4] Che R. X. Wang Y. F. Li K. X. Xu Z. H. Hu J. M. Wang F.. (2019). Degraded patch formation significantly changed microbial community composition in alpine meadow soils. Soil Till Res. 195:104426. 10.1016/j.still.2019.104426

[B5] Chen B. X. Zhang X. Z. Tao J. Wu J. S. Wang J. S. Shi P. L.. (2014). The impact of climate change and anthropogenic activities on alpine grassland over the Qinghai-Tibet Plateau. Agric. Forest Meteorol. 189, 11–18. 10.1016/j.agrformet.2014.01.00236751969

[B6] Chen D. D. Sun D. S. Zhang S. H. Tan Y. R. Du G. Z. Shi X. M. (2011). Effect of grazing intensity on soil microbial characteristics of an alpine meadow on the Tibetan plateau. J. Lanzhou Univ. Nat. Sci. 47, 73–77.36733589

[B7] Chen J. Xiao Q. C. Xu D. L. Li Z. S. Chao L. M. Li X. Y.. (2023). Soil microbial community composition and co-occurrence network responses to mild and severe disturbances in volcanic areas. Sci. Total Environ. 901:165889. 10.1016/j.scitotenv.2023.165889 37524180

[B8] Cheng J. A. Jin H. Xu Z. X. Yang X. Y. Qin B. Zhang J. L. (2021). Effects of degraded plant *Stellera chamaejasme* L. on the rhizosphere soil microbial communities in typical alpine grassland, Gansu Province. Acta Microbiol. Sinica. 61, 3686–3704.

[B9] Cheng J. A. Jin H. Zhang J. L. Xu Z. X. Yang X. Y. Liu H. Y.. (2022). Effects of allelochemicals, soil enzyme activities, and environmental factors on rhizosphere soil microbial community of L. along a growth-coverage gradient. Microorganisms 10:158. 10.3390/microorganisms10010158 35056607 PMC8781187

[B10] Cheng Z. Chen Y. Zhang F. (2018). Effect of reclamation of abandoned salinized farmland on soil bacterial communities in arid northwest China. Sci. Total Environ. 630, 799–808. 10.1016/j.scitotenv.2018.02.259 29494981

[B11] Dai Z. M. Guo X. Lin J. H. Wang X. He D. Zeng R. J.. (2023). Metallic micronutrients are associated with the structure and function of the soil microbiome. Nat. Commun. 14:8456. 10.1038/s41467-023-44182-2 38114499 PMC10730613

[B12] de Vries F. T. Griffiths R. I. Bailey M. Craig H. Girlanda M. Gweon H. S.. (2018). Soil bacterial networks are less stable under drought than fungal networks. Nat. Commun. 9:3033. 10.1038/s41467-018-05516-7 30072764 PMC6072794

[B13] Dong S. K. Shang Z. H. Gao J. X. Boone R. B. (2020). Enhancing sustainability of grassland ecosystems through ecological restoration and grazing management in an era of climate change on Qinghai-Tibetan Plateau. Agric. Ecosyst. Environ. 287:106684. 10.1016/j.agee.2019.106684

[B14] Draganova D. Valcheva I. Kuzmanova Y. Naydenov M. (2018). Effect of wheat straw and cellulose degrading fungi of genus Trichoderma on soil respiration and cellulase betaglucosidase and soil carbon content. Agric. Sci. Technol. 10, 349–353. 10.15547/10.15547/ast.2018.04.064

[B15] Du X. F. Yu H. Wang S. Deng Y. (2019). Metagenomics reveal responses of soil microbial community in grassland to global changes. Chin. J. Ecol.. S 38, 3516–3526.

[B16] Feng J. Wei K. Chen Z. H. Lü X. T. Tian J. H. Wang C.. (2019). Coupling and decoupling of soil carbon and nutrient cycles across an aridity gradient in the drylands of northern China: evidence from ecoenzymatic stoichiometry. Global Biogeochem. Cycles. 33, 559–569. 10.1029/2018GB006112

[B17] Franklin R. B. Mills A. L. (2003). Multi-scale variation in spatial heterogeneity for microbial community structure in an eastern Virginia agricultural field. FEMS Microbiol. Ecol. 44, 335–346. 10.1016/S0168-6496(03)00074-6 12830827

[B18] Gao X. L. Lv S. H. Diao Z. Y. Wang D. W. Li D. K. Zheng Z. R. (2023). Responses of vegetation, soil, and microbes and carbon and nitrogen pools to semiarid grassland land-use patterns in Duolun, Inner Mongolia, China. Sustainability 15:3434. 10.3390/su15043434

[B19] Han Q. Q. Chen Y. H. Li Z. C. Zhang Z. Qin Y. O. Liu Z. K.. (2024). Changes in the soil fungal communities of steppe grasslands at varying degradation levels in North China. Can. J. Microbiol. 70, 70–85. 10.1139/cjm-2023-0105 38096505

[B20] Han X. Li Y. H. Du X. F. Li Y. B. Wang Z. W. Jiang S. W.. (2020). Effect of grassland degradation on soil quality and soil biotic community in a semi-arid temperate steppe. Ecol. Proc. 9, 1–11. 10.1186/s13717-020-00256-3

[B21] Huang J. S. Hu B. Qi K. B. Chen W. J. Pang X. Y. Bao W. K.. (2016). Effects of phosphorus addition on soil microbial biomass and community composition in a subalpine spruce plantation. Eur. J. Soil Biol. 72, 35–41. 10.1016/j.ejsobi.2015.12.007

[B22] Huo A. D. Wang X. Zhao Z. X. Yang L. Y. Zhong F. Q. Zheng C. L.. (2022). Risk Assessment of heavy metal pollution in farmland soils at the northern foot of the Qinling Mountains, China. Int. J. Environ. Res. Public Health 19:14962. 10.3390/ijerph192214962 36429680 PMC9690618

[B23] Jaklitsch W. M. Lechat C. Voglmayr H. (2014). The rise and fall of Sarawakus (Hypocreaceae, Ascomycota). Mycologia 106, 133–144. 10.3852/13-117 24603837 PMC4140013

[B24] Jiang M. F. Liu J. Y. Sun H. R. Chen Q. B. Jin H. Yang J. Y.. (2024). Soil microbial diversity and composition response to degradation of the alpine meadow in the southeastern Qinghai-Tibet Plateau. Environ. Sci. Pollut. Res. 31, 26076–26088. 10.1007/s11356-024-32536-2 38491240

[B25] Jin H. Cheng J. A. Liu H. Y. Yang X. Y. Dai L. Huang X. C.. (2024). Characterization of the microbial community structures, soil chemical properties, and enzyme activity of *Stellera chamaejasme* (Thymelaeaceae) and its associated forages in alpine grassland of Northwestern China. Curr. Microbiol. 81:39. 10.1007/s00284-023-03554-z 38097817

[B26] Jin Z. Zhong W. Wu S. Han C. (2018). Effect of vegetation degradation on microbial communities in alpine grassland soils in Northwest Yunnan. Acta Microbiol. Sin. 58, 2174–2185. 10.13343/j.cnki.wsxb.20180039

[B27] Kotzé E. Sandhage-Hofmann A. Amelung W. Oomen R. J. du Preez C. C. (2017). Soil microbial communities in different rangeland management systems of a sandy savanna and clayey grassland ecosystem, South Africa. Nutr. Cycling Agroecosyst. 107, 227–245. 10.1007/s10705-017-9832-3

[B28] Kumaresan D. Stephenson J. Doxey A. C. Bandukwala H. Brooks E. Hillebrand-Voiculescu A.. (2018). Aerobic methylotrophs in Movile Cave: genomic and metagenomic analyses. Microbiome 6, 1–10. 10.1186/s40168-017-0383-2 29291746 PMC5748958

[B29] Legay N. Baxendale C. Grigulis K. Krainer U. Kastl E. Schloter M.. (2014). Contribution of above- and below-ground plant traits to the structure and function of grassland soil microbial communities. Ann. Bot. 114, 1011–1021. 10.1093/aob/mcu169 25122656 PMC4171078

[B30] Li C. M. Zhang D. R. Xu G. C. Yan R. Huang Y. Feng L. Q.. (2023). Effects of alpine grassland degradation on soil microbial communities in Qilian Mountains of China. J. Soil Sci. Plant Nutr. 23, 912–923. 10.1007/s42729-022-01092-4

[B31] Li H. Y. Qiu Y. Z. Yao T. Han D. R. Gao Y. M. Zhang J. G.. (2021a). Nutrients available in the soil regulate the changes of soil microbial community alongside degradation of alpine meadows in the northeast of the Qinghai-Tibet Plateau. Sci. Total Environ. 792:148363. 10.1016/j.scitotenv.2021.148363 34465051

[B32] Li H. Y. Yao T. Gao Y. M. Zhang J. G. Ma Y. C. Lu X. W.. (2019). Relationship between soil fungal community and soil environmental factors in degraded alpine grassland. Acta Microbiol. Sin. 59, 678–688. 10.13343/j.cnki.wsxb.20180257

[B33] Li H. Y. Yao T. Zhang J. G. Gao Y. M. Ma Y. C. Lu X. W.. (2018). Relationship between soil bacterial community and environmental factors in the degraded alpine grassland of eastern Qilian Mountains, China. Chinese J. Appl. Ecol. 29, 3793–3801. 10.13287/j.1001-9332.201811.037 30460826

[B34] Li J. Awasthi M. K. Zhu Q. Chen X. Y. Wu F. Q. Wu F. Y.. (2021b). Modified soil physicochemical properties promoted sequestration of organic and inorganic carbon synergistically during revegetation in desertified land. J. Environ. Chem. Eng. 9:106331. 10.1016/j.jece.2021.106331

[B35] Li J. G. Pu L. J. Zhu M. Zhang J. Li P. Dai X. Q.. (2014). Evolution of soil properties following reclamation in coastal areas: a review. Geoderma 226, 130–139. 10.1016/j.geoderma.2014.02.003

[B36] Li Y. C. Li Y. F. Chang S. X. Liang X. Qin H. Chen J. H.. (2017). Linking soil fungal community structure and function to soil organic carbon chemical composition in intensively managed subtropical bamboo forests. Soil Biol. Biochem. 107, 19–31. 10.1016/j.soilbio.2016.12.024

[B37] Lin B. Zhao X. Zheng Y. Qi S. Liu X. (2017). Effect of grazing intensity on protozoan community, microbial biomass, and enzyme activity in an alpine meadow on the Tibetan Plateau. J. Soils Sediments. 17, 2752–2762. 10.1007/s11368-017-1695-3

[B38] Liu C. S. Zhao D. F. Ma W. J. Guo Y. D. Wang A. J. Wang Q. L.. (2016). Denitrifying sulfide removal process on high-salinity wastewaters in the presence of *Halomonas* sp. Appl. Microbiol. Biotechnol. 100, 1421–1426. 10.1007/s00253-015-7039-6 26454867

[B39] Liu D. Song X. Y. Liu Y. Wang C. T. (2023). Effects of phosphorus application on soil phosphorus forms and phoD-harboring microbial communities in an alpine grassland on the Qinghai-Tibetan Plateau. Front. Ecol. Evolution. 11:1131408. 10.3389/fevo.2023.1131408

[B40] Luo S. Png G. K. Ostle N. J. Zhou H. K. Hou X. Y. Luo C. L.. (2023). Grassland degradation-induced declines in soil fungal complexity reduce fungal community stability and ecosystem multifunctionality. Soil Biol. Biochem. 176:108865. 10.1016/j.soilbio.2022.108865

[B41] Luo Z. M. Liu J. X. Jia T. Chai B. F. Wu T. H. (2020). Soil bacterial community response and nitrogen cycling variations associated with subalpine meadow degradation on the Loess Plateau, China. Appl. Environ. Microbiol. 86, e00180–e00120. 10.1128/AEM.00180-20 32144107 PMC7170489

[B42] Ma X. W. Ren B. H. Yu J. X. Wang J. Y. Bai L. Li J. H.. (2023). Changes in grassland soil types lead to different characteristics of bacterial and fungal communities in Northwest Liaoning, China. Front. Microbiol. 14:1205574. 10.3389/fmicb.2023.1205574 37448571 PMC10336218

[B43] Magoc T. Salzberg S. L. (2011). FLASH: fast length adjustment of short reads to improve genome assemblies. Bioinformatics 27, 2957–2963. 10.1093/bioinformatics/btr507 21903629 PMC3198573

[B44] Mahvi A. H. Eslami F. Baghani A. N. Khanjani N. Yaghmaeian K. Mansoorian H. J. (2022). Heavy metal pollution status in soil for different land activities by contamination indices and ecological risk assessment. Int. J. Environ. Sci. Technol. 19, 7599–7616. 10.1007/s13762-022-03960-z

[B45] Masuda Y. Mise K. Xu Z. X. Zhang Z. C. Shiratori Y. Senoo K.. (2024). Global soil metagenomics reveals distribution and predominance of in nitrogen-fixing microbiome. Microbiome. 12:95. 10.1186/s40168-024-01812-1 38790049 PMC11127431

[B46] McGuire K. L. Bent E. Borneman J. Majumder A. Allison S. D. Treseder K. K. (2010). Functional diversity in resource use by fungi. Ecology 91, 2324–2332. 10.1890/09-0654.1 20836454

[B47] Miguel B. Manuel D. Santiago S. Rocío H. Yanchuang Z. J G. J.. (2020). Global ecosystem thresholds driven by aridity. Science 367, 787–790. 10.1126/science.aay5958 32054762

[B48] Mukhopadhya I. Hansen R. El-Omar E. M. Hold G. L. (2012). IBD-what role do Proteobacteria play? Nat. Rev. Gastroenterol. Hepatol. 9, 219–230. 10.1038/nrgastro.2012.14 22349170

[B49] Murugan R. Loges R. Taube F. Sradnick A. Joergensen R. G. (2014). Changes in soil microbial biomass and residual indices as ecological indicators of land use change in Temperate Permanent Grassland. Microb. Ecol. 67, 907–918. 10.1007/s00248-014-0383-8 24549746

[B50] Nian L. L. Zhang X. N. Li L. L. Zhou S. Y. D. Liu X. Y. Li X. D.. (2024). Effects of alpine meadows with different degradation gradients on the stability of the soil micro-foodweb in the Tibetan Plateau. Ecol. Indic. 158:111390. 10.1016/j.ecolind.2023.111390

[B51] Pan H. Liu H. Y. Liu Y. W. Zhang Q. C. Luo Y. Liu X. M.. (2018). Understanding the relationships between grazing intensity and the distribution of nitrifying communities in grassland soils. Sci. Total Environ. 634, 1157–1164. 10.1016/j.scitotenv.2018.04.117 29660872

[B52] Peng F. Xue X. Li C. Lai C. Sun J. Tsubo M.. (2020a). Plant community of alpine steppe shows stronger association with soil properties than alpine meadow alongside degradation. Sci. Total Environ. 733:139048. 10.1016/j.scitotenv.2020.139048 32446054

[B53] Peng F. Xue X. You Q. G. Sun J. Zhou J. Wang T.. (2020b). Change in the trade-off between aboveground and belowground biomass of alpine grassland: implications for the land degradation process. Land Degr. Dev. 31, 105–117. 10.1002/ldr.3432

[B54] Reiss K. C. Brown M. T. Lane C. R. (2010). Characteristic community structure of Florida's subtropical wetlands: the Florida wetland condition index for depressional marshes, depressional forested, and flowing water forested wetlands. Wetlands Ecol. Manage. 18, 543–556. 10.1007/s11273-009-9132-z

[B55] Ren Y. Yu G. Shi C. P. Liu L. M. Guo Q. Han C.. (2022). Majorbio Cloud: a one-stop, comprehensive bioinformatic platform for multiomics analyses. Imeta 1:e12. 10.1002/imt2.12 38868573 PMC10989754

[B56] Ren Z. Wang Z. M. Wang Y. Ma P. P. Niu D. C. Fu H.. (2021). Soil bacterial communities vary with grassland degradation in the Qinghai Lake watershed. Plant Soil. 460, 541–557. 10.1007/s11104-020-04823-7

[B57] Sterkenburg E. Bahr A. Durling M. B. Clemmensen K. E. Lindahl B. D. (2015). Changes in fungal communities along a boreal forest soil fertility gradient. New Phytol. 207, 1145–1158. 10.1111/nph.13426 25952659

[B58] Su Y. Z. Li Y. L. Zhao H. L. (2006). Soil properties and their spatial pattern in a degraded sandy grassland under post-grazing restoration, inner Mongolia, northern China. Biogeochemistry 79, 297–314. 10.1007/s10533-005-5273-1

[B59] Thomson B. C. Ostle N. McNamara N. Bailey M. J. Whiteley A. S. Griffiths R. I. (2010). Vegetation affects the relative abundances of dominant soil bacterial taxa and soil respiration rates in an upland grassland soil. Microb. Ecol. 59, 335–343. 10.1007/s00248-009-9575-z 19705192

[B60] Wang B. H. Ma Z. H. Fu W. L. (2008a). Determination of heavy metal in soil by high pressure sealed vessels assisted digestion-atomic absorption spectrometry. Trans. Chin. Soc. Agric. Eng. 24, 265–269.

[B61] Wang D. J. Zhou H. K. Zuo J. Chen P. She Y. D. Yao B. Q.. (2022). Responses of soil microbial metabolic activity and community structure to different degraded and restored grassland gradients of the Tibetan Plateau. Front. Plant Sci. 13:770315. 10.3389/fpls.2022.770315 35463442 PMC9024238

[B62] Wang M. C. Liu Y. H. Wang Q. Gong M. Hua X. M. Pang Y. J.. (2008b). Impacts of methamidophos on the biochemical, catabolic, and genetic characteristics of soil microbial communities. Soil Biol. Biochem. 40, 778–788. 10.1016/j.soilbio.2007.10.012

[B63] Wang M. J. Liu M. X. Wang Q. Y. Xiao Y. D. (2023). Soil-microbe characterization and interaction in alpine degraded grassland in Maqu county. China Environ. Sci. 43, 6482–6489.

[B64] Wang Y. Ren Z. Ma P. Wang Z. Niu D. Fu H.. (2020). Effects of grassland degradation on ecological stoichiometry of soil ecosystems on the Qinghai-Tibet Plateau. Sci. Total Environ. 722:137910. 10.1016/j.scitotenv.2020.137910 32192971

[B65] Xiong W. Zhao Q. Zhao J. Xun W. Li R. Zhang R.. (2015). Different continuous cropping spans significantly affect microbial community membership and structure in a vanilla-grown soil as revealed by deep pyrosequencing. Microb. Ecol. 70, 209–218. 10.1007/s00248-014-0516-0 25391237

[B66] Xue Y. Y. Zhang B. Q. He C. S. Shao R. (2019). Detecting vegetation variations and main drivers over the agropastoral ecotone of Northern China through the ensemble empirical mode decomposition method. Remote Sensing. 11:1860. 10.3390/rs11161860

[B67] Yang Z. Li X. Y. Yuan Y. Yu H. Jinxi L. (2023). Characteristics and influence factors of aoil urease in a typical monsoon evergreen broad-leaved forest in Southern Yunnan. Forest Grassland Resour. Res. 35, 71–79. 10.13466/j.cnki.lyzygl.2023.04.009

[B68] Yao J. N. Liu S. Zhang J. J. Hu M. Z. Dai J. X. (2024). Enzyme activity and microbial metabolic diversity in shrub rhizosphere soil in Ningxia desert steppe. Acta Pratac. Sinica. 33, 1–14. 10.11686/cyxb2023380

[B69] Ye H. Zhao Y. He S. L. Wu Z. D. Yue M. Hong M. (2024). Metagenomics reveals the response of desert steppe microbial communities and carbon-nitrogen cycling functional genes to nitrogen deposition. Front. Microbiol. 15:1369196. 10.3389/fmicb.2024.1369196 38596372 PMC11002186

[B70] Yu Y. Zheng L. Zhou Y. J. Sang W. G. Zhao J. N. Liu L.. (2021). Changes in soil microbial community structure and function following degradation in a temperate grassland. J. Plant Ecol. 14, 384–397. 10.1093/jpe/rtaa102

[B71] Zeng Q. C. An S. S. Liu Y. (2017). Soil bacterial community response to vegetation succession after fencing in the grassland of China. Sci. Total Environ. 609, 2–10. 10.1016/j.scitotenv.2017.07.102 28732294

[B72] Zhang H. J. Chen W. L. Dong L. Z. Wang W. (2024). Grassland degradation amplifies the negative effect of nitrogen enrichment on soil microbial community stability. Glob. Chang. Biol. 30:e17217. 10.1111/gcb.17217 38456565

[B73] Zhou H. Zhang D. G. Jiang Z. H. Sun P. Xiao H. L. Wu Y. X.. (2019). Changes in the soil microbial communities of alpine steppe at Qinghai-Tibetan Plateau under different degradation levels. Sci. Total Environ. 651, 2281–2291. 10.1016/j.scitotenv.2018.09.336 30326458

[B74] Zhou H. C. Yao Y. J. Liang T. Zhang Y. Q. Zhang D. J. Sun B.. (2020). Risk of heavy metal pollution in soil of alpine meadow with different degradation gradients in Tianzhu county. Ecol. Environ. Sci. 29, 2102–2109. 10.16258/j.cnki.1674-5906.2020.10.021

