# Supplementary material for: Effects of grassland degradation on soil ecological stoichiometry and soil microbial community on the South of the Greater Khingan Mountains
Source: Front Microbiol. 2024 Nov 18;15:1438787. doi: 10.3389/fmicb.2024.1438787 (PMC11610252; doi:10.3389/fmicb.2024.1438787)
Supplement: Supplementary file 1 [file Table_1.docx]

**Supplementary data**

Effects of grassland degradation on soil ecological stoichiometry and soil microbial community on the South of the Greater Khingan Mountains

Yuyu Li^1, 2^, Lixing Zhao^3^, Mian Gul Hilal^1^, Lizhu Guo^1, 2^, Yandong Zhang^1^, Yu Ji^1, 2^, Xiaowei Jiang^1^, Lifen Hao^1, 2,^* and Kejian Lin^1, 2,^*

*^1^ Institute of Grassland Research, Chinese Academy of Agricultural Science, Hohhot 010010, China*

*^2^ Key Laboratory of Biohazard Monitoring, Green Prevention and Control for Artificial Grassland, Ministry of Agriculture and Rural Affairs, Hohhot 010010, China*

*^3^ Hinggan League Institute of Agricultural and Husbandry Sciences, Hinggan 137400, China*

*Correspondence: linkejian@caas.cn (K.L.); haolifen@caas.cn (L.H.)

**TABLE S1** Description of the basic situation of grassland degradation on the south of the Greater Khingan Mountains. NDG, non-degraded grasslands; LDG, lightly degraded grasslands; MDG, moderately degraded grasslands; SDG, severely degraded grasslands.

| Sample plot | Longitude, latitude | Altitude (m) | Main vegetation | Vegetation coverage (%) |
| --- | --- | --- | --- | --- |
| NDG | 120°09´24´´E, 45°52´56´´N | 984 | *Carex pediformis*, Filifolium sibiricum L., Chrysanthemum naktongense,Stipa baicalensis | >85% |
| LDG | 120°08´27´´E, 45°53´52´´N | 950 | *Carex pediformis*, Ixeris denticulata,Stipa baicalensis,Cleistogenes squarrosa | 55-85% |
| MDG | 120°08´06´´E, 45°54´36´´N | 910 | *Carex pediformis*, Chrysanthemum naktongense, Stipa baicalensis, Ixeris denticulata | 25-55% |
| SDG | 120°02´39´´E, 46°03´42´´N | 861 | *Cleistogenes squarrosa*, Carex pediformis, Stipa baicalensis, Stellera chamaejasme L., | <25% |

NDG, non-degraded grasslands; LDG, lightly degraded grasslands; MDG, moderately degraded grasslands; SDG, severely degraded grasslands.

**Fig. S1.** Field photos of grasslands with different degrees of degradation (photos taken by Lixing Zhao). NDG, non-degraded grasslands; LDG, lightly degraded grasslands; MDG, moderately degraded grasslands; SDG, severely degraded grasslands.


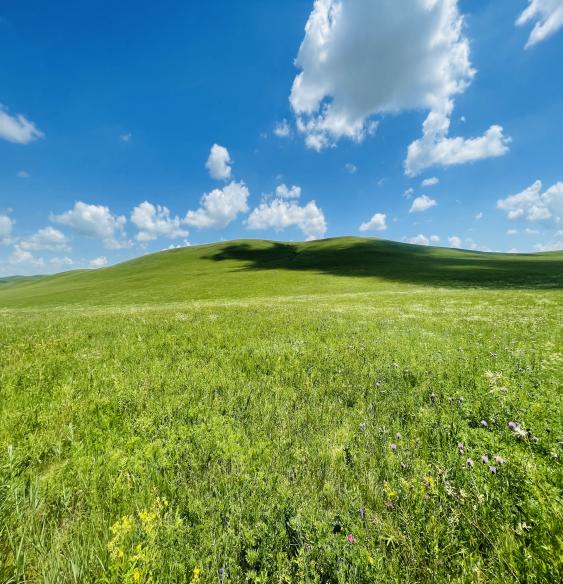

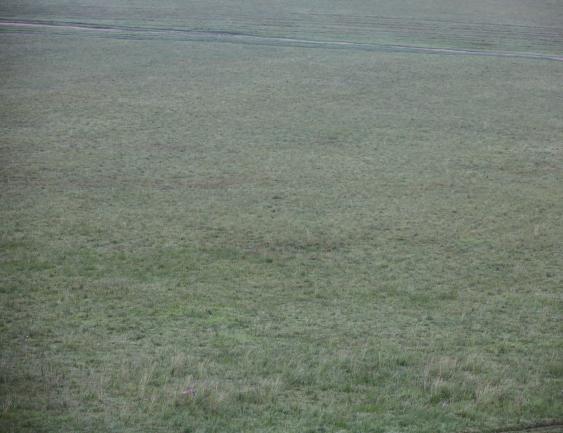

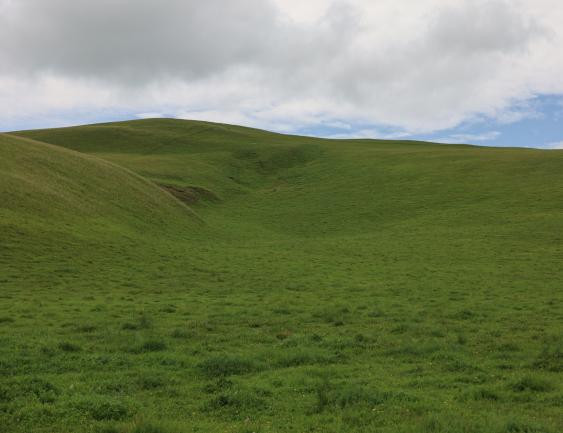

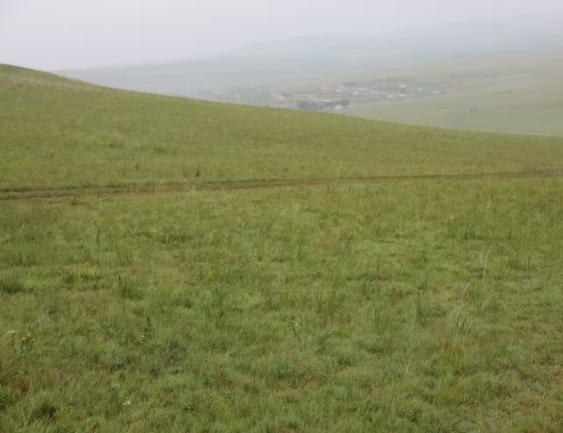


**NDG**

**LDG**

**MDG**

**SDG**
